# Bibliometric and visualized analysis of exercise and osteoporosis from 2002 to 2021

**DOI:** 10.3389/fmed.2022.944444

**Published:** 2022-12-08

**Authors:** Fan Li, Weixin Xie, Yi Han, Zhanchun Li, Jie Xiao

**Affiliations:** ^1^Department of Orthopedic Surgery, Renji Hospital, School of Medicine, Shanghai Jiao Tong University, Shanghai, China; ^2^Department of Anesthesiology, Renji Hospital, School of Medicine, Shanghai Jiao Tong University, Shanghai, China

**Keywords:** exercise, osteoporosis, bibliometrics, visualization analysis, citespace, VOSviewer

## Abstract

**Background:**

Bibliometric analysis was designed to investigate a systematic understanding of developments in exercise and osteoporosis research over the past 20 years.

**Methods:**

Relevant publications from the Web of Science Core Collection were downloaded on April 26, 2022. CiteSpace, VOSviewer, and the online bibliometric analysis platform were used to conduct this scientometric study.

**Results:**

A total of 5518 publications were in 1202 academic journals with 137405 co-cited references in by 5637 institutions from 98 countries/regions. The country leading the research was the USA. The University of Melbourne was the most active institution. Osteoporosis International was the most productive journal concerning exercise and osteoporosis research. According to the burst references, “low-level vibration,” “high-frequency” and “resistance exercise” have been recognized as the hotspots research in the domain. The keywords co-occurrence analysis identified “skeletal muscle,” “sarcopenia” and “mesenchymal stem cell” as the important future research directions.

**Conclusion:**

This study was the first comprehensive metrological and statistical analysis of exercise and osteoporosis research over the past 20 years. Our findings would provide guidance to understand the research frontiers and hot directions in the near future.

## Introduction

Osteoporosis is defined as a systemic skeletal disorder characterized by low bone mass and deterioration of the skeletal microarchitecture and subsequent increased risk of bone fragility and fracture, thus contributing to disability and mortality. Low bone mineral density (BMD) is one of the most important characters of osteoporosis. According to the Global Burden of Disease (GBD) Study 2019,^1^ the global burden of low BMD increases remarkably over time, low BMD and subsequent osteoporotic fracture has become the important cause of disability-adjusted life years (1). Despite significant advances in osteoporosis treatment with pharmacological interventions, many individuals at high risk do not receive adequate treatment (2). Widespread implementation of effective prevention remains the best option for the future.

Over the years, there has been a growing interest in the benefits of exercise for people with osteoporosis. At adolescence, physical exercise is clearly an effective way to increase the peak bone mass (3). A sedentary lifestyle, little exercise, promotes the loss of bone mass (4). Exercising regularly can improve BMD (5). In addition, exercise can build muscle strength and improve body balance, which reduces the risk of falling and fractures from osteoporosis (6, 7). According to the clinical guideline developed by different countries and institutions, exercise is strongly recommended for the treatment of osteoporosis (8–10). Various exercise therapies are used to improve bone health, but there is no clear evidence to conclusively support the superiority of any particular exercise intervention. Although the field of exercise and osteoporosis has produced a great number of scholarly documents, there are no studies that gather bibliometric data in a systematic manner to identify the research hotspots and frontiers of this field.

Bibliometrics is a quantitative statistical tool used to analyze published literature. It has been applied to assess citation counts and collaboration in countries, institutions, journals, and authors, and to predict keyword trends of research activities over time (11). In recent years, some bibliometric researches of exercise have mentioned the benefit of improving bone mass, such as tai chi (12) and yoga (13). However, there is no specific bibliometric analysis of exercise intervention and osteoporosis. In this study, we intended to analyze the research trends, hotspots and research frontiers in the field of exercise and osteoporosis. The results of this work will be helpful for further research in this field.

## Materials and methods

### Data acquisition and search strategy

**FIGURE 1:**
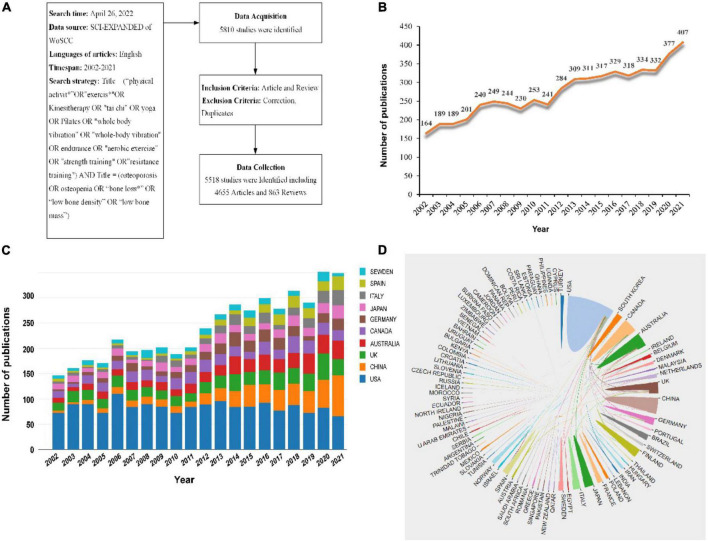
**(A)** The search strategy. **(B)** The annual number of publications on the topic of exercise and osteoporosis research from 2002 to 2021. **(C)** The annual number of publications in the top 10 countries from 2002 to 2021. **(D)** The collaboration analysis among different countries/regions from 2002 to 2021.

Full records and cited references of articles were downloaded from the Web of Science Core Collection (WoSCC) database, which included Social Science Citation (SCI)-EXPANDED. A complete online search was carried out on 26 April 2022. Our search strategy was as follows (Figure 1A): Title = (“exercis*” OR “physical activit*” OR kinesitherapy OR “tai chi” OR yoga OR pilates OR “whole body vibration” OR “whole-body vibration” OR endurance OR “aerobic exercise” OR “strength training” OR “resistance training”) AND Title = (osteoporosis OR osteopenia OR “bone loss*” OR “low bone density” OR “low bone mass”) AND Language = English. We choose the time span of 2002-2021 as our search dates. Only peer-reviewed published original articles or reviews were included.

### Data collection

Two investigators (FL and WX) independently conducted the primary search. Before reaching a consensus, any disagreements were discussed. The collected data included publication counts, titles, authors, countries, institutions, journals, highly cited articles and keywords. The WoSCC data were converted to txt format and imported into CiteSpace V5.8.R3 SE, 64bit (Drexel University, Philadelphia, PA, USA), VOSviewer 1.6.18 (Leiden University, Leiden, The Netherlands) and the Online Analysis Platform of Literature Metrology^2^ for further bibliometric analysis. The impact factor (IF) and Quartile in category of journals were obtained from the 2021 Journal Citation Reports (Clarivate Analytics, Philadelphia, PA, USA).

### Bibliometric analysis

CiteSpace is usually applied to visualize and analyze the publications. In our research, CiteSpace was utilized to, respectively, identify the productive authors, institutions, co-cited authors, co-cited references, references and keywords with the strongest citation bursts, as well as the related visual networks. In the networks, links between nodes represent the relationship of cooperation or co-citations and the different node size and colors represent different items and years, respectively (14). Besides, nodes with purple rings indicate high betweenness centrality, which are usually identified as hotspots or turning points in the field (15). VOSviewer is also used to construct visualization networks (16). In this study, VOSviewer was utilized to visualize keywords co-occurrence and journal co-citation networks. In addition, the online analysis platform was applied to identify the partnership and annual publication outputs of countries/regions. Additionally, to further identify research frontiers in this field, we searched relevant clinical studies that had been registered at ClinicalTrials.gov. The annual publication trend was represented by Microsoft Excel 2019 (Microsoft Corporation, Redmond, WA, USA).

## Results

### Annual growth trend of publications

The systematic search identified 5518 publications met the inclusion criteria between 2002 and 2021, including 4655 articles and 863 reviews. The annual publication trend was shown in Figure 1B. As can be seen, despite the appearance of the volatility to decrease and stagnate, the number of related publications increased steadily over time, with most published in 2021 (*n* = 407), indicating that exercise and osteoporosis research has become a hotspot and captures global research attention now.

### Countries/Regions and institutions analysis

To identify productive countries/regions and institutions leading the research in this field, further analysis of publications in different countries and institutions was conducted. The top 10 countries/regions and institutions were shown in Figure 1C and Table 1 according to the publication number, and the top 5 countries were: USA, United Kingdom, China, Australia and Canada. The bar chart presented that the USA dominated in this field in terms of publications counts prior to 2021, which achieved the peak about 110 in 2006. However, the number of publications in China experienced steady growth since 2011, and even surpassed the USA for the first time in 2021. As everyone knows, the Chinese government is increasingly investing in health care. The sustained economic growth also promotes scientific research output. Next, we analyzed the international cooperation among different countries. The line between the two countries indicated the existence of cooperation. It can be seen that the most frequent collaboration was the USA, followed by England, Australia and Canada (Figure 1D). The USA mainly cooperated closely with Canada, Australia and China, while China largely cooperated with USA and England.

**TABLE 1:** The top 10 countries/regions and institutions in exercise and osteoporosis research between 2002 and 2021.

Rank	Country/Region	Count	Institution	Count
1	USA (North America)	1701	Univ Melbourne (Australia)	132
2	England (Europe)	461	Univ Toronto (Canada)	100
3	China (Asia)	441	Univ British Columbia (Canada)	94
4	Australia (Oceania)	441	Harvard Univ (USA)	90
5	Canada (North America)	405	Univ Calif San Francisco (USA)	76
6	Germany (Europe)	299	McMaster Univ (Canada)	75
7	Japan (Asia)	294	Univ Pittsburgh (USA)	72
8	Italy (Europe)	257	Deakin Univ (Australia)	66
9	Spain (Europe)	216	Chinese Univ Hong Kong (China)	55
10	Sweden (Europe)	213	Sweden Lund Univ (Sweden)	55

**FIGURE 2:**
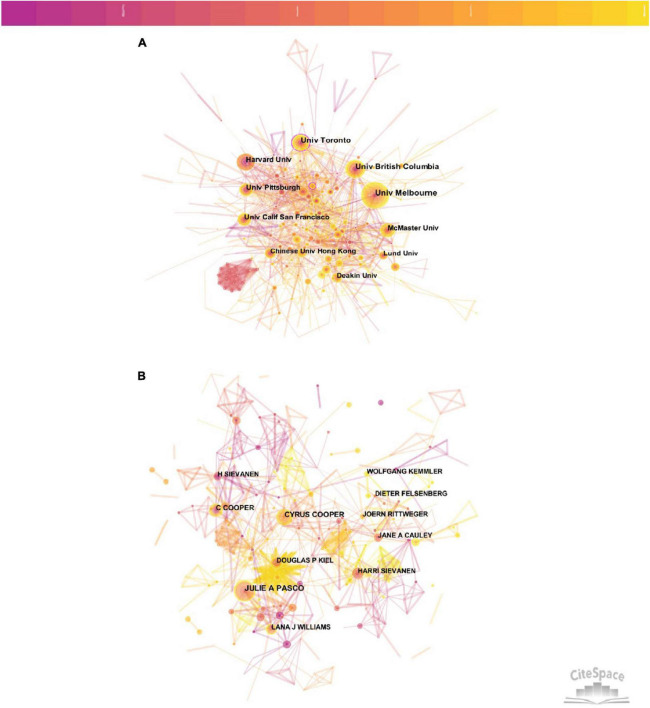
**(A)** Visualization map of co-institution analysis by using CiteSpace software. **(B)** Visualization map of author co-citation analysis by using CiteSpace software.

The top 10 institutions were distributed in five countries. Of these, three were from the Canada, three were from USA, two were from Australia and the remaining two were from China and Sweden. A total of two institutions published more than 100 papers: Univ Melbourne and Univ Toronto (Table 1). The merged co-institution network map was shown in Figure 2A, with 573 nodes and 1640 links. The line thickness indicates the strength of cooperation. A network density of only 0.01 indicated that the cooperation between these institutions was not close enough.

### Authors and co-cited authors

The number of publications produced by one author is able to represent the degree of research activity and contribution in the field. As shown in Table 2, three authors published over 30 articles. Cyrus Cooper published the most papers (*n* = 53) and ranked first, followed by Harri Sievanen (*n* = 40) and Julie A Pasco (*n* = 32). In most cases, cross-cooperation can promote productivity of a certain study. The merged co-authorship network map is shown in Figure 2B, including 1288 nodes and 2929 links. The node sizes of Cyrus Cooper and Harri Sievanen were larger due to the fact of having more publications. Close cooperation was observed among several authors, such as Julie A Pasco and Lana J Williams, Wolfgang Kemmler and Simon Von Stengel, etc. The co-cited authors network was visualized by Supplementary Figure 1. The most prominent nodes were linked to the most co-cited authors, five authors had co-citations over 400 (Table 2). Kanis JA had the most co-citations (*n* = 979) and ranked the first, followed by Cummings SR (*n* = 677), Frost HM (*n* = 475), Cyrus Cooper (*n* = 415) and Turner CH (*n* = 401).

**TABLE 2:** The top 10 authors and co-cited authors in exercise and osteoporosis research between 2002 and 2021.

Rank	Author	Count	Co-cited author	Count
1	Cyrus Cooper	53	Kanis JA	979
2	Harri Sievanen	40	Cummings SR	677
3	Julie A Pasco	32	Frost HM	475
4	Douglas P Kiel	20	Cyrus Cooper	415
5	Lana J Williams	19	Turner CH	401
6	Jane A Cauley	19	Heaney RP	396
7	Joern Rittweger	18	Melton LJ	391
8	Wolfgang Kemmler	17	Johnell O	381
9	Dieter Felsenberg	17	Seeman E	380
10	Simon Von Stengel	16	Riggs BL	331

### Analysis of journals and co-cited journals

**FIGURE 3:**
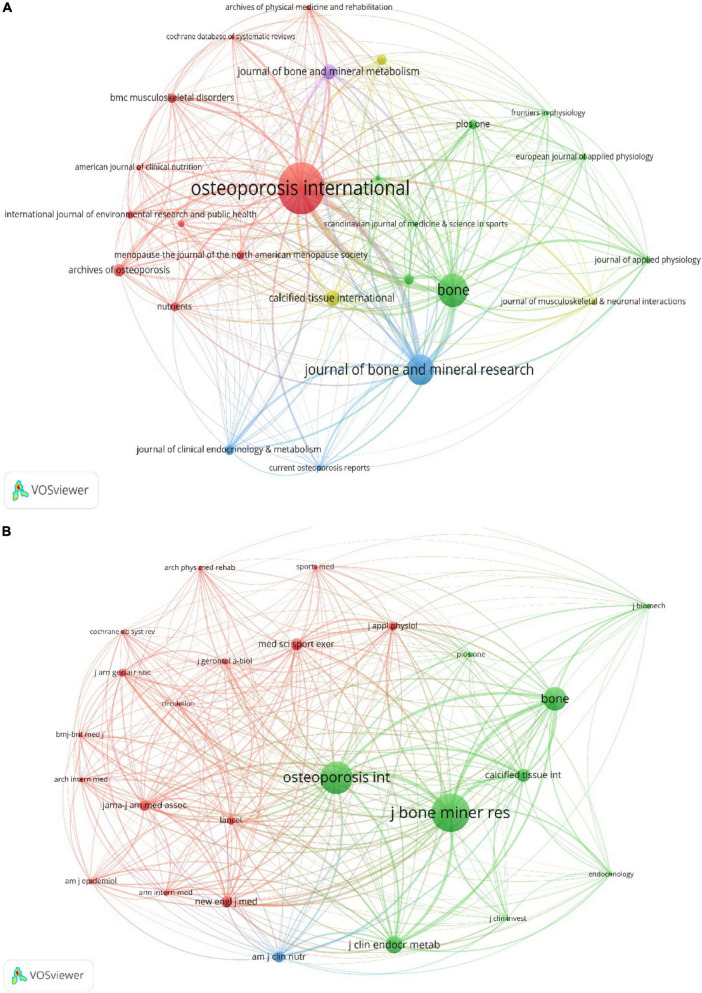
**(A)** Visualization map of journals analysis by using VOSviewer software. **(B)** Visualization map of journal co-citation analysis by using VOSviewer software.

A total of 5518 publications were published in 1202 academic journals. The top 20 most prolific and co-cited journals were listed in Supplementary Tables 1, 2. Since two journals had the same number of publications and shared the tenth place, there were a total of 21 journals. Of these top 20 journals, the majority of these active journals were from the USA. Notably, there was no Chinese journal in the two tables, indicating that China should attach great importance to the development of international journals and further improve the academic influence in this field. Over 200 papers were published in three journals, Osteoporosis International (*n* = 438, 7.94%, IF = 4.507) published the most documents in this field, followed by Bone (*n* = 257, 4.66%, IF = 4.398) and Journal of Bone and Mineral Research (*n* = 230, 4.17%, IF = 6.741). Among the top 20 most prolific journals, Journal of Bone and Mineral Research had the highest IF. Furthermore, eleven journals were classified as Q2 and five were classified as Q1. As for the research domain of these journals, nine were classified into orthopedics. Figure 3A showed that Osteoporosis International had active citation relationships with Bone and Journal of Bone and Mineral Research. The most frequently co-cited journal was Journal of Bone and Mineral Research (3964 citations). Among the top 20 co-cited journals, New England Journal of Medicine (1929 citations) had the highest IF of 91.253. As shown in Figure 3B, Journal of Bone and Mineral Research had strong co-cited relationships with Osteoporosis International, Bone and Journal of Clinical Endocrinology and Metabolism.

### Analysis of co-cited references

**FIGURE 4:**
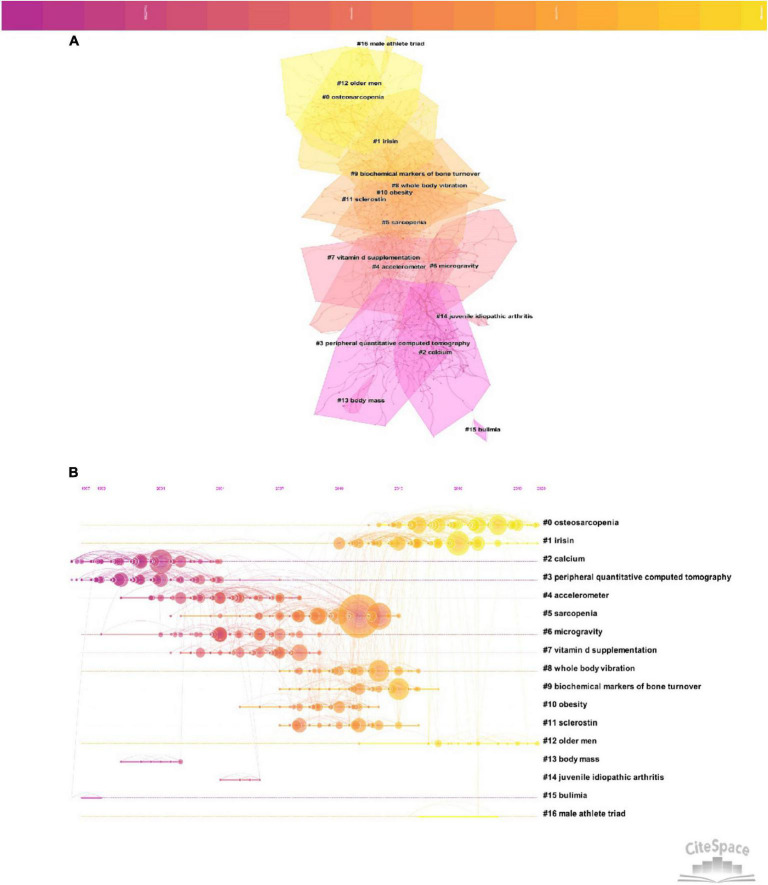
**(A)** The clustered network map of co-cited references by using CiteSpace software. **(B)** The timeline view of co-citation clusters by using CiteSpace software.

The co-citation and clustered network map were identified by CiteSpace from 137405 references in a hierarchical order. There were 908 nodes and 1983 links in the network map (Figure 4A). The network map was divided into several different clusters (*Q* = 0.7205, *S* = 0.8842). It can be seen that 17 major cluster labels were marked, including osteosarcopenia, irisin, calcium, peripheral quantitative computed tomography, accelerometer, sarcopenia, microgravity, vitamin d supplementation, whole body vibration, biochemical markers of bone turnover, obesity, sclerostin, older men, body mass, juvenile idiopathic arthritis, bulimia and male athlete triad. Furthermore, we also provided the timeline view for the major clusters in Figure 4B, from which the evolution characteristics of each cluster was displayed clearly. We found that prior to 2007, calcium and peripheral quantitative computed tomography were relatively early hotspots. Whole body vibration and biochemical markers of bone turnover were mid-term research hotspots (2007–2014). The recent research seemed to focus on osteosarcopenia and irisin. The top 10 cited articles were demonstrated in Table 3. Six studies were published between 2012 and 2019. The highest number of citations was Howe, TE in Cochrane Database of Systematic Reviews (100 times), followed by Weaver, CM in Osteoporosis International (84 times) and Klibanski, A in JAMA- Journal of the American Medical Association (67 times).

**TABLE 3:** The top 10 high-cited papers in exercise and osteoporosis research between 2002 and 2021.

Rank	Title	Journal	First author	Publication year	Frequency
1	Exercise for preventing and treating osteoporosis in postmenopausal women	Cochrane Database of Systematic Reviews	Howe, TE	2011	100
2	The National Osteoporosis Foundation’s position statement on peak bone mass development and lifestyle factors: a systematic review and implementation recommendations	Osteoporosis International	Weaver, CM	2016	84
3	Osteoporosis prevention, diagnosis, and therapy	JAMA- Journal of the American Medical Association	Klibanski, A	2001	67
4	Interventions for preventing falls in older people living in the community	Cochrane Database of Systematic Reviews	Gillespie, LD	2012	63
5	Clinician’s Guide to Prevention and Treatment of Osteoporosis	Osteoporosis International	Cosman, F	2014	54
6	Effects of training on bone mass in older adults: a systematic review	Sports Medicine	Gomez-Cabello, A	2012	49
7	European guidance for the diagnosis and management of osteoporosis in postmenopausal women	Osteoporosis International	Kanis, JA	2013	46
8	High-Intensity Resistance and Impact Training Improves Bone Mineral Density and Physical Function in Postmenopausal Women with Osteopenia and Osteoporosis: The LIFTMOR Randomized Controlled Trial	Journal of Bone and Mineral Research	Watson, SL	2018	45
9	Targeted exercise against osteoporosis: A systematic review and meta-analysis for optimizing bone strength throughout life	BMC Medicine	Nikander, R	2010	43
10	FRAX (TM) and the assessment of fracture probability in men and women from the UK	Osteoporosis International	Kanis, JA	2008	41

Analyzing recent registered trials can provide us with the new information of research frontiers. We especially extracted the related 16 trials that had been registered at ClinicalTrials.gov between 2020 and 2022 (Supplementary Table 3). The clinical trial numbered NCT05541432 focused on the effect of different types of exercise intensity on BMD. The clinical trial numbered NCT04380155 believed that there is a threshold of time where continued stimulus can touch off an additional metabolic response in bone. Although the bone benefit observed with higher intensity resistance training or impact exercise for bone, not all elderly people can do it. Based on the global vibration or body vibration through vibrating platforms, the clinical trial numbered NCT05538377 will explore the effect of focal vibration devices on bone. In addition, some trials are currently identifying the mechanisms of exercise on osteoporosis. The clinical trial numbered NCT05541432 thought that bone marrow fat may play an important role in the mechanisms. The clinical trials numbered NCT04815824 and NCT04275011 focused on the disruptions in calcium homeostasis during different mode of exercise and the level of sclerostin in the blood, respectively. Furthermore, the bone benefit of exercise will be exploring in pediatric cancer survivors and people with special disease, such as chronic kidney disease and systemic lupus erythematosus, which are described in trials numbered NCT05392790, NCT04271605 and NCT04472286. In terms of detection mode, the clinical trial numbered NCT05060380 assessed BMD by magnetic resonance imaging (MRI) technique.

### Analysis of keyword co-occurrence

**FIGURE 5:**
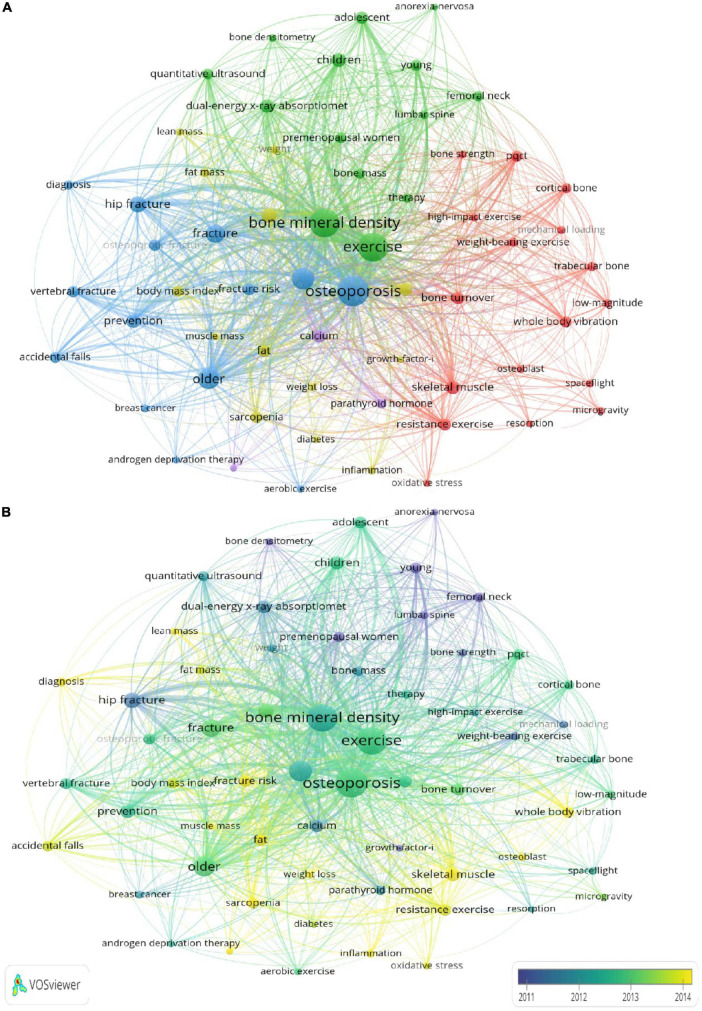
**(A)** Visualization map of keywords co-occurrence analysis by using VOSviewer software. **(B)** The average publication year.

By analyzing the contents in the titles and abstracts of the included manuscripts, VOSviewer identified 61 keywords with a minimum occurrence of 60 times (Figure 5A). On VOSviewer visualization, the number of occurrences was represented by the size of the bubble and the cluster was identified by the specific color. The co-occurrence analysis of high-frequency keywords clarified the leading hotspots in this field. The red cluster focused on the type of exercise, such as whole-body vibration, resistance exercise and weight-bearing exercise, as well as the molecular mechanisms of exercise on osteoporosis. The yellow cluster focused on the association of body composition and osteoporosis. The green cluster concentrated on the detection methods of osteoporosis, including dual-energy x-ray absorptiometry and quantitative ultrasound, as well as the key population of osteoporosis. The blue cluster mainly concentrated that the correlation of exercise and fracture. Over time, research attention had gradually shifted to changes in body composition, resistance exercise and skeletal muscle (Figure 5B).

### References and keywords burst analysis

**FIGURE 6:**
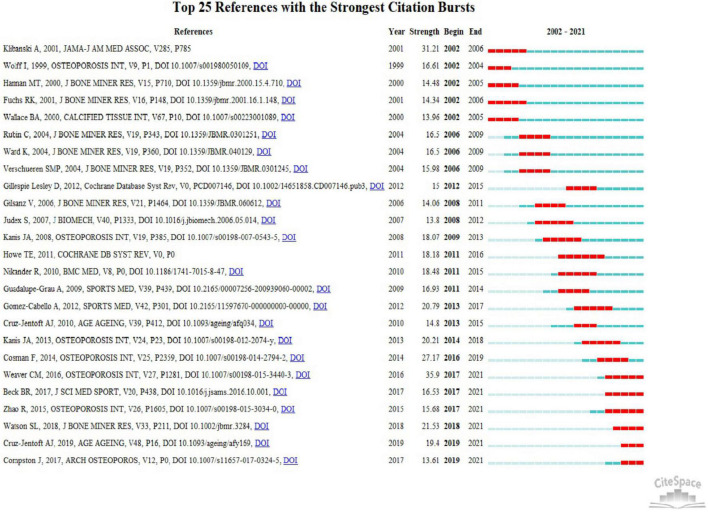
The top 25 references with the strong citation bursts between 2002 and 2021.

We used CiteSpace to detect emergent references to identify the topics that were actively discussed in this field. The red lines indicated the time period when the references or keywords burst occurred, and the blue lines represented the time interval. Figure 6 illustrated the top 25 references with the high citation outbreaks, and the burst strength of these references ranged from 13.61 to 35.9. The strongest burst (*n* = 35.9) was caused by the paper entitled “The National Osteoporosis Foundation’s position statement on peak bone mass development and lifestyle factors: a systematic review and implementation recommendation” authored by Weaver et al. (8) with citation burst from 2017 to 2021. This study systematically described the influence of exercise on peak bone mass. Moreover, the burst in the majority of references was over, but the burst in several references is still ongoing, indicating that these topics have attracted continuous attention in recent years.

**FIGURE 7:**
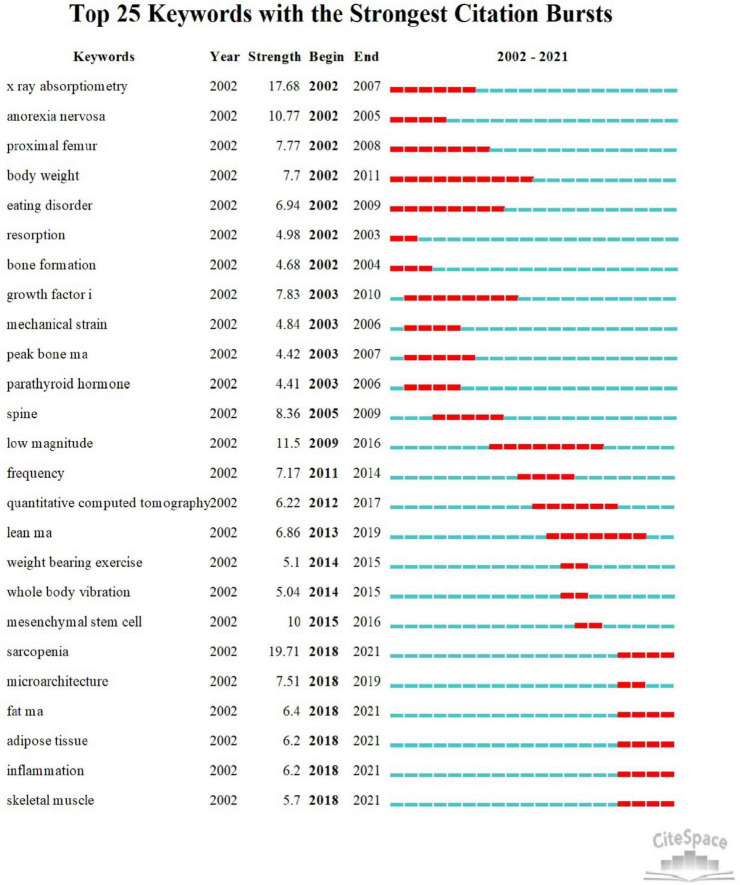
The top 25 keywords with the strong citation bursts between 2002 and 2021.

The top 25 keywords with citation bursts were presented in Figure 7. Keywords “sarcopenia” with the strongest burst (*n* = 19.71) appeared in 2018, followed by “x ray absorptiometry” (*n* = 17.68), indicating the importance of skeletal muscle of osteoporosis. The recent keywords with citation bursts occurred in 2018 were “inflammation,” “adipose tissue,” “skeletal muscle” and “fat mass.” Furthermore, these keywords also continued to 2021.

## Discussion

This study visualized research articles in exercise and osteoporosis research from 2002 to 2021. A total of 5518 papers were in 1202 academic journals with 137405 co-cited references in by 5637 institutions from 98 countries/regions. The annual output of the publications was slowly increasing over the past 20 years, indicating that an increase in researcher interest in this field. In the 1970s, Aloia et al. (17) observed that exercise could prevent involutional bone loss. Researchers around the world pay more attention to the effectiveness of specific types and durations of exercise. However, overall analysis of the research status, development tendencies, and hotspots had been lacking. In the past two decades, the focus of exercise and osteoporosis research has changed gradually in various aspects, including different types of exercise interventions, different ages and populations, the techniques for measuring BMD and the relevant mechanism. Our study analyzed publications about exercise and osteoporosis research from multiple dimensions, and showed a systematic view to understand this field.

Adequate nutrition is identified as a strategy to counteract osteoporosis, particularly with regard to vitamin D, calcium and protein intake. It is necessary to achieve adequate nutrition for maximizing the skeletal benefits of exercise.

Exercise is generally recognized as an important mechanical stimulus for the development and maintenance of bone health and quality, but not all exercise interventions exert positive effect on bone mass. As seen from the clusters of co-cited publications and keyword analysis, the researches of exercise interventions were largely concentrated in aerobic exercise, whole-body vibration (WBV), high-impact exercise, weight-bearing exercise and resistance exercise. Astronauts experience more bone loss per month than the loss on Earth in postmenopausal women per year (18). Due to reduced weight-bearing, microgravity and complete bed rest induce the bone loss. Most of the studies that investigated the effect of aerobic exercise on BMD is carried out with walking. Consistently, walking provides a modest increase in the loads, which have little or no effect on bone mass. A hotspot of concern from 2007 to 2012 was WBV exercise, which provides high-frequency mechanical stimuli. Several RCT studies found WBV exercise increased BMD of femoral neck significantly in postmenopausal women (19–21). There were five references with citation burst between 2006 and 2012, which were about low-magnitude and high-frequency exercise. These prospective trials (19, 20, 22, 23) demonstrated that low-level vibration significantly prevented bone loss in postmenopausal women, disabled children and young women. Another highly cited article showed that the effect of WBV exercise on BMD is influenced by the different frequency, amplitude, time course and the type of vibration exercise, such as vertical or rotational form of vibration (24). These differences found between studies prevent researchers from exploring the ideal WBV interventions. The results of top keywords using burst detection showed that weight bearing exercise attracted most of the attention of researchers from 2014 to 2015. In 2014, the guideline (10), developed by the National Osteoporosis Foundation, recommended weight-bearing and muscle-strengthening exercise to maintain or improve bone strength. RCTs and meta-analyses consistently agreed that the most effective type of exercise for increasing BMD of femoral neck was non-weight bearing high force exercise, such as progressive resistance strength training for the lower limbs. For BMD at the spine, the most beneficial intervention was combination exercise, which was the combination of weight bearing and non-weight bearing exercise in a training programme (25). The combination of resistance exercise and high-impact or weight-bearing exercise can significantly improve BMD (26, 27). Therefore, one of the current challenges in this field is developing multi-component exercises to efficiently maintain and improve the bone mass in different age groups.

It is worth mentioning that osteoporotic fracture, a major complication of osteoporosis, is associated with substantial morbidity and mortality. The clustered network map of keywords analysis reflected the relationship between fracture and exercise. The majority of non-vertebral fractures occur from a fall, there is strong evidence that exercise could reduce falls, which primarily involve balance and functional exercises (28). Although exercise has beneficial effects on BMD and fall, there is a paucity of direct evidence to support fracture prevention efficacy of exercise (29). For those individuals at high risk of fracture, high-impact exercises also increase the risk of fracture. To confirm the exercise intervention trial for osteoporotic fracture prevention, a sample size of thousands of individuals at high risk of low trauma fracture will be required in the future.

Another trend in exercise and osteoporosis research is the shift from postmenopausal women to different age groups and other populations with diseases. To our knowledge, the majority of previous research is clinical trials that focused on postmenopausal women. The results of this bibliometric analysis indicate that exercise is recommended for the prevention of osteoporosis at all stages of life, such as children, young, adolescent and old people. The skeleton of growing children is particularly sensitive to exercise, exercise in adolescence and early adulthood can significantly increase peak bone mass (30). An interesting study reported that a 10% higher peak bone mass was able to delay the development of osteoporosis by 13 years (31). Overall, exercise interventions for maintaining bone mass should begin in childhood and continue throughout life. In addition to postmenopausal osteoporosis, many diseases develop with bone loss. The keywords of “sarcopenia” had the highest burst strength. Sarcopenia often co-occurs with osteoporosis, which is characterized by low bone density and loss of muscle mass. The European Working Group on Sarcopenia in Older People (32) suggested that physical exercise was very effective for counteracting sarcopenia and improving bone mass and muscle mass. Low BMD is one of the major complications of eating disorder (33), duration of weight-bearing exercise may be protective of BMD in anorexia nervosa patients (34). Besides, exercise therapy is also recommended for the treatment of other diseases, which develop with bone loss, such as breast cancer (35), diabetes (36) and prostate cancer patients on androgen deprivation therapy (37).

Mechanical loading from physical exercise imposes an anabolic stimulus on bone. The mechanical signal was observed to promote both proliferation of mesenchymal stem cell (MSC) and capacity for osteogenic differentiation (38). Recently, the molecular mechanisms of exercise on osteoporosis may lead to a new understanding. The “growth factor i,” “MSC,” “adipose tissue” and “inflammation” had relatively high citation bursts. The skeletal muscle secretes various molecules that affect bone including insulin-like growth factor-1 (IGF-1), basic fibroblast growth factor (FGF-2), interleukin-6 (IL-6), IL-15 and myostatin (39). Furthermore, irisin secreted by skeletal muscle was considered to improve bone health following exercise (40). Adipocytes also produce leptin, adiponectin and IL-6, which potentially modulate bone metabolisms.

A wide variety of techniques are available to evaluate BMD. The most widely used are dual energy X-ray absorptiometry (DXA), which only measures bone mass and areal BMD. However, there are many technical limitations in the application of DXA for measurements (41) and the results are unremarkable sometimes. Compared to DXA, peripheral quantitative computed tomography (pQCT) provides direct measures of bone geometry and volumetric BMD. In recent years, the short-term change in BMD is also evaluated by the test of surrogate markers of bone turnover in the blood or urine, but it is insensitive to long-term monitoring. Therefore, future work should expand the detection methods of BMD in order to comprehensively understand the changes of bone mass following exercise.

### Strengths and limitations

To the best of our knowledge, our study based on bibliometric tools, including CiteSpace, VOSviewer, and the online bibliometric analysis platform, analyzed the knowledge base and research hotspots on exercise and osteoporosis research. However, this study had several limitations. The data were only retrieved from the WoSCC database. The clinical trials were only searched from ClinicalTrials.gov. In addition, only English documents were considered. Therefore, the data may not be comprehensive enough.

## Conclusion

Our study firstly provides a bibliometric analysis of exercise and osteoporosis research from the publications over the past 20 years. According to our findings, the foci of research on exercise and osteoporosis has changed in multiple dimensions, including different types of exercise interventions, different age groups, the related mechanism and the method for measuring BMD. High-impact exercises or resistance exercises combined with high-impact training have a more beneficial effect that should be promoted in a preventative approach for improving bone mass. Further work is required to explore the ideal exercise interventions at different stages of life. The crosstalk impact between skeletal muscle and bone following exercise provides a new method for the mechanisms governing bone mass in the context of exercise. Overall, researchers could clearly understand the fundamental knowledge, and benefit from the analysis of research hotspots and frontiers. Our results will provide helpful clues for future research directions and scientific strategies in this field.

## Data availability statement

The original contributions presented in this study are included in the article/Supplementary material, further inquiries can be directed to the corresponding authors.

## Author contributions

FL and WX designed the study. FL and YH drafted the manuscript. JX revised the manuscript. FL and ZL analyzed the data. All authors contributed to the article and approved the submitted version.

## Footnotes

1
http://ghdx.healthdata.org/gbd-results-tool

2
http://bibliometric.com/

## Supplementary material

Supplementary Figure 1 Visualization map of co-cited authors analysis by using CiteSpace software.

Supplementary Table 1 The top 20 journals of exercise and osteoporosis research between 2002 and 2021.

Supplementary Table 2 The top 20 co-cited journals of exercise and osteoporosis research between 2002 and 2021.

Supplementary Table 3 Sixteen clinical trials exploring exercise and osteoporosis research that were registered with ClinicalTrials.gov.
