# Supplementary figures and images for: Bibliometric and visualized analysis of exercise and osteoporosis from 2002 to 2021
Source: Front Med (Lausanne). 2022 Dec 8;9:944444. doi: 10.3389/fmed.2022.944444 (PMC9773261; doi:10.3389/fmed.2022.944444)

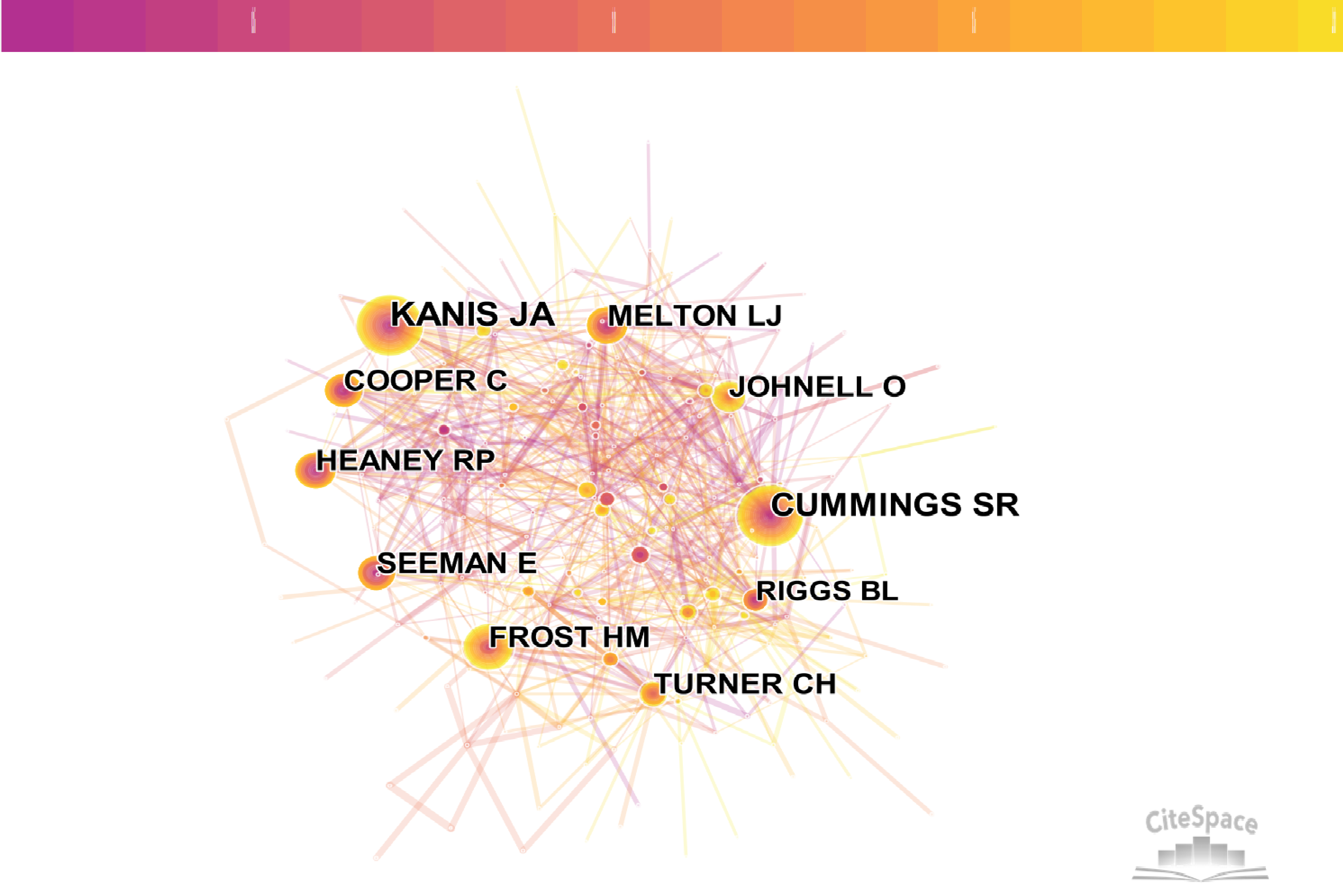

Supplement: Supplementary Figure 1 — Visualization map of co-cited authors analysis by using CiteSpace software. [file Image_1.jpeg]
