# Supplementary material for: Bibliometric and visualized analysis of exercise and osteoporosis from 2002 to 2021
Source: Front Med (Lausanne). 2022 Dec 8;9:944444. doi: 10.3389/fmed.2022.944444 (PMC9773261; doi:10.3389/fmed.2022.944444)
Supplement: Supplementary Table 1 — The top 20 journals of exercise and osteoporosis research between 2002 and 2021. [file Table_1.pdf]

Supplementary Table 1 | The top 20 journals of exercise and osteoporosis research between 2002 and 2021.

Rank	Journal	Country	Count	Impact factor (2020)	Quartile in category (2020)
1	Osteoporosis International	ENGLAND	438	4.507	Q2
2	Bone	USA	257	4.398	Q2
3	Journal of Bone and Mineral Research	USA	230	6.741	Q1
4	Calcified Tissue International	USA	102	4.333	Q2
5	Journal of Bone and Mineral Metabolism	JAPAN	100	2.626	Q4
6	Archives of Osteoporosis	ENGLAND	78	2.617	Q4
7	Journal of Clinical Densitometry	USA	67	2.617	Q4
8	Medicine and Science in Sprots and Exercise	USA	64	5.411	Q1
9	Journal of Clinical Endocrinology & Metabolism	USA	62	5.958	Q1
10	Plos One	USA	58	3.240	Q2
11	BMC Musculoskeletal Disorders	ENGLAND	57	2.355	Q3
12	Menopause-the Journal of the North American Menopause Society	USA	53	2.953	Q2
13	Nutrients	SWITZERLAND	53	5.719	Q1
14	International Journal of Environmental Research and Public Health	SWITZERLAND	47	3.39	Q2
15	Maturitas	NETHERLANDS	40	4.342	Q2
16	Journal of Applied Physiology	USA	35	3.532	Q2
17	Journal of Musculoskeletal & Neuronal Interactions	GREECE	35	2.041	Q4
18	Current Osteoporosis Reports	USA	33	5.096	Q2
19	European Journal of Applied Physiology	GERMANY	33	3.078	Q2
20	Aging Clinical and Experimental Research	ITALY	31	3.638	Q3
21	American Journal of Clinical Nutrition	USA	31	7.047	Q1
