# Supplementary material for: Bibliometric and visualized analysis of exercise and osteoporosis from 2002 to 2021
Source: Front Med (Lausanne). 2022 Dec 8;9:944444. doi: 10.3389/fmed.2022.944444 (PMC9773261; doi:10.3389/fmed.2022.944444)
Supplement: Supplementary Table 2 — The top 20 co-cited journals of exercise and osteoporosis research between 2002 and 2021. [file Table_2.DOCX]

| Rank | Co-cited Journal | Country | Co-citation | Impact factor  (2021) | Quartile  in category (2021) |
| --- | --- | --- | --- | --- | --- |
| 1 | Journal of Bone and Mineral Research | USA | 3964 | 6.741 | Q1 |
| 2 | Osteoporosis International | ENGLAND | 3940 | 4.507 | Q2 |
| 3 | Bone | USA | 3314 | 4.398 | Q2 |
| 4 | Calcified Tissue International | USA | 2535 | 4.333 | Q2 |
| 5 | Journal of Clinical Endocrinology & Metabolism | USA | 2361 | 5.958 | Q1 |
| 6 | JAMA- Journal of the American Medical Association | USA | 1954 | 56.274 | Q1 |
| 7 | New England Journal of Medicine | USA | 1929 | 91.253 | Q1 |
| 8 | Lancet | ENGLAND | 1805 | 79.323 | Q1 |
| 9 | Medicine and Science in Sports and Exercise | USA | 1803 | 5.411 | Q1 |
| 10 | American Journal of Clinical Nutrition | USA | 1529 | 7.047 | Q1 |
| 11 | Journal of Applied Physiology | USA | 1212 | 3.532 | Q2 |
| 12 | Annals of Internal Medicine | USA | 1199 | 25.391 | Q1 |
| 13 | Journal of the American Geriatrics Society | USA | 1191 | 5.562 | Q1 |
| 14 | Journal of Bone and Mineral Metabolism | JAPAN | 1096 | 2.626 | Q4 |
| 15 | American Journal of Epidemiology | USA | 1086 | 4.897 | Q1 |
| 16 | Archives of Internal Medicine | USA | 998 | / | / |
| 17 | Journal of Clinical Densitometry | USA | 985 | 2.617 | Q4 |
| 18 | Journal of Gerontology Series A-Biological Sciences and Medical Sciences | USA | 971 | 6.053 | Q1 |
| 19 | Plos One | USA | 962 | 3.240 | Q2 |
| 20 | BMJ-British Medical Journal | ENGLAND | 879 | 39.89 | Q1 |

**Supplementary Table 2 |** The top 20 co-cited journals of exercise and osteoporosis research between 2002 and 2021.
