# Supplementary material for: Bibliometric and visualized analysis of exercise and osteoporosis from 2002 to 2021
Source: Front Med (Lausanne). 2022 Dec 8;9:944444. doi: 10.3389/fmed.2022.944444 (PMC9773261; doi:10.3389/fmed.2022.944444)
Supplement: Supplementary Table 3 — Sixteen clinical trials exploring exercise and osteoporosis research that were registered with ClinicalTrials.gov. [file Table_3.pdf]

Supplementary Table 3 | Sixteen clinical trials exploring exercise and osteoporosis research that were registered with ClinicalTrials.gov.

NCT number	Study title	Status	Conditions	Interventions	Sample size (n)	Study type	Study design
NCT05541432	Finding the Optimal Resistance Training Intensity For Your Bones	Recruiting	Osteoporosis<br>Osteopenia	Other: Supervised strength training (group 1)<br>Other: Supervised strength training (group 2)<br>Other: Home exercise	324	Interventional	Allocation: Randomized<br>Intervention Model: Parallel Assignment<br>Masking: Triple (Participant, Investigator, Outcomes Assessor)<br>Primary Purpose: Treatment
NCT05316272	Effects of DHEA and Exercise on Bone Marrow Fat in Postmenopausal Women	Enrolling by invitation	Low Bone Density<br>Osteoporosis	Behavioral: Exercise<br>Dietary Supplement: DHEA	52	Interventional	Allocation: Randomized<br>Intervention Model: Parallel Assignment<br>Masking: Triple (Participant, Investigator, Outcomes Assessor)<br>Primary Purpose: Prevention
NCT05060380	Feasibility of a Novel Resistance Exercise in Individuals With Osteoporosis	Not yet recruiting	Osteopenia<br>Osteoporosis<br>Postmenopausal Osteoporosis<br>Sarcopenia	Other: Exercise training	44	Interventional	Allocation: Randomized<br>Intervention Model: Parallel Assignment<br>Masking: Single (Outcomes Assessor)<br>Primary Purpose: Other

NCT05538377	Effect of Focal Vibration Within a Multicomponent Exercise Program for Older Women With Osteoporosis a Single-blind Clinical Trial	Not yet recruiting	Osteoporosis Osteoporosis, Postmenopausal	Other: Focal Vibration Other: Control Group	34	Interventional	Allocation: Randomized Intervention Model: Parallel Assignment Masking: Single (Outcomes Assessor) Primary Purpose: Treatment
NCT04815824	Exercise Mode and Bone	Recruiting	Osteoporosis Aging	Behavioral: Exercise Mode	60	Interventional	Allocation: Randomized Intervention Model: Crossover Assignment Masking: None (Open Label) Primary Purpose: Prevention
NCT04345250	Bone Response to Exercise and Energy Restriction in Young Adults	Not yet recruiting	Bone Resorption Bone Loss	Other: Energy restriction	12	Interventional	Allocation: N/A Intervention Model: Single Group Assignment Masking: None (Open Label) Primary Purpose: Prevention
NCT04380155	Cycling Duration and Bone Markers in in Active Young Adults	Suspended	Bone Loss Energy Supply; Deficiency	Other: Exercise	12	Interventional	Allocation: N/A Intervention Model: Single Group Assignment Masking: None (Open Label) Primary Purpose: Prevention

NCT05266976	Mode of Exercise and Bone Biomarkers in Older Veterans	Recruiting	Aging<br>Musculoskeletal Diseases<br>Osteoporosis	Behavioral: 10 Weeks of Supervised Resistance Exercise Training<br>Behavioral: 10 Weeks of Supervised Endurance Exercise Training	120	Interventional	Allocation: Randomized<br>Intervention Model: Parallel Assignment<br>Masking: None (Open Label)<br>Primary Purpose: Prevention
NCT05392790	Progressive Resisted Exercise Plus Aerobic Exercise on Osteoporotic Systemic Lupus Erythmatosus	Recruiting	Systemic Lupus Erythematosus	Other: Progressive resisted exercise training<br>Drug: Calcium and Vit D<br>Other: Aerobic exercises	80	Interventional	Allocation: Randomized<br>Intervention Model: Parallel Assignment<br>Masking: Double (Participant, Investigator)<br>Primary Purpose: Treatment
NCT04275011	Bone Response to Exercise in Women on Antiresorptive Medications	Terminated	Osteoporosis<br>Bone Loss	Other: Progressive Resistance and Impact Exercise<br>Other: Static Balance and Postural Exercise	26	Interventional	Allocation: Randomized<br>Intervention Model: Parallel Assignment<br>Masking: Double (Investigator, Outcomes Assessor)<br>Primary Purpose: Other
NCT04653350	High Intensity Multi-Modal Exercise Training in Postmenopausal Women	Completed	Postmenopausal Osteoporosis<br>Postmenopausal Osteopenia	Other: High Intensity Exercises<br>Other: General Exercises	58	Interventional	Allocation: Randomized<br>Intervention Model: Parallel Assignment<br>Masking: Double (Participant, Outcomes Assessor)<br>Primary Purpose: Treatment

NCT04168658	A Physical Activity Program for Female Nursing Home Residents at Risk of Osteoporosis	Not yet recruiting	Osteoporosis	Combination Product: Physical activity and education intervention<br>Behavioral: Education intervention	80	Interventional	Allocation: Randomized<br>Intervention Model: Parallel Assignment<br>Masking: Single (Outcomes Assessor)<br>Primary Purpose: Prevention
NCT04271605	Intervention on Osteoporosis and Chronic Kidney Disease-mineral and Bone Disorder (CKD-MBD)	Terminated	Osteoporosis, Postmenopausal	Behavioral: diet and exercise suggestion	5	Interventional	Allocation: N/A<br>Intervention Model: Single Group Assignment<br>Masking: None (Open Label)<br>Primary Purpose: Treatment
NCT04444661	Effects of COVID-19 Induced Deconditioning After Long-term High Intensity Resistance Exercise	Completed	Osteoporosis<br>Sarcopenia	/	21	Observational	Observational Model: Cohort<br>Time Perspective: Retrospective
NCT04472286	Healthy Bones, Healthy Life	Recruiting	Acute Lymphoblastic Leukemia, Pediatric<br>Pediatric ALL<br>Pediatric Lymphoma<br>(and 10 more...)	/	50	Observational	Observational Model: Cohort<br>Time Perspective: Prospective
NCT04076618	Incorporating Nutrition, Vests, Education, and Strength Training in Bone Health (INVEST in Bone Health)	Recruiting	Weight Loss<br>Bone Health	Device: Vest<br>Behavioral: Resistance Exercise Training<br>Behavioral: weight loss program	192	Interventional	Allocation: Randomized<br>Intervention Model: Parallel Assignment<br>Masking: Double (Investigator, Outcomes Assessor)<br>Primary Purpose: Prevention
